# District of Columbia

**Published:** 1898-05

**Authors:** J. P. Turner

**Affiliations:** Secretary


### VETERINARY MEDICAL ASSOCIATION OF THE DISTRICT OF COLUMBIA.

THE regular bimonthly meeting (the seventeenth) was called to order at Elks' Hall, 1006 E Street, N. W., on January 29th, by the Vice-President, Dr. Buckingham. Members present: Drs. French, Robinson (C. B.), Buckingham, Barton, Walmer, Turner, Salmon, C. F. Hadfield, R. H. Hadfield, and Yetton.

Unfinished business was the report of the Legislative Committee on the bill introduced in Congress to regulate the practice of veterinary medicine in the District of Columbia. The committee reported that it had drawn a suitable bill, and that it was before the District Committee at that time.

Drs. Barton and Walmer were appointed a committee to draw up suitable resolutions in memory of our late fellow-member, Dr. Adamson, who was recently killed while in practice at Minneapolis.

A paper was read by the Secretary regarding the workings of the Newark (N. J.) Milk Company.

Dr. C. B. Robinson made an interesting report on his inspection of the cattle and dairy of the Pasteur Milk Company, a company in which the District Medical Society is largely interested.

Dr. Salmon gave a very interesting and profitable address on the meat-inspection methods of the Agricultural Department, following which was a general discussion on the subject of meat-inspection.

Dr. C. B. Robinson then reported a disease discovered and named by him as "sonus neurosis." This disease is found only among horses serving in the fire department, and has been under observation for several years. During the last two weeks four cases were observed. The symptoms are manifested upon the ringing of the gong in the station or hospital, or even by a pistol-crack, or the sudden slamming of a door. Immediately following any of these noises the animal gets excited, and a spasmodic contraction of the muscles of the leg will be observed, either fore- or hind-leg may be equally affected. The leg will frequently be elevated to an angle of forty-five degrees and held there some time. Ringing the gong continuously increases the symptoms. The lameness does not persist, as the animal warms out of it. When cases are taken to the hospital they usually resolve in a few days, but frequently re-attacks occur and several horses have been transferred to other work than answering fire-alarms, owing to its persistency. At other work these symptoms are not observed. Dr. Robinson's theory is that of auditory irritation.

The President appointed Drs. French and Walmer to prepare papers for the next meeting.

Upon motion of Dr. Salmon, the meeting adjourned.

The regular bimonthly meeting (the eighteenth) was called to order at Elks' Hall, March 26, 1898, by the President, Dr. Acheson. The following members responded to the roll-call: Drs. Acheson, Barton, Buckingham, R. A. Hadfield, Pearson, C. B. Robinson, Turner, and Yetton. Visitor: Dr. Robertson, veterinarian, U. S. Army.

The Legislative Committee, through its chairman, Dr. Buckingham, reported its inability to get a hearing before the District Committees of Congress.

The Committee on Resolutions appointed to draw up fitting resolutions relative to our recent loss by death of Dr. John H. Adamson, reported the following resolutions:

WHEREAS, It has pleased Almighty God to remove from his earthly labor our fellow-member, Dr. John H. Adamson; and

WHEREAS, This Association deeply grieves the loss of Dr. Adamson, who by his genial disposition, manly traits, and high professional attainments, has endeared himself to all of us; be it therefore

*Resolvd*, That a copy of these resolutions be spread on the minutes of this Association and a copy be forwarded to each of the veterinary journals and to the family of our deceased member.

The election of officers to serve for the coming year resulted in a re-election of the present officers: President, Dr. Acheson; Vice-President, Dr. Buckingham; Secretary and Treasurer, Dr. Turner; Trustee for three years, Dr. C. B. Robinson; Trustee for two years, Dr. Pearson; Trustee for one year, Dr. Walmer.

The papers which were to have been read at this meeting by Drs. Walmer and French were continued over to the next meeting.

Dr. C. B. Robinson reported another case of "sonus neurosis" which has been under the observation of several of our members. In speaking further about this disease, Dr. Robinson stated that during his connection with the fire department of the District, covering the last fifteen years, he had seen more than fifty cases of this disease, but had never reported them, supposing the disease had been observed abroad; but after a recent search of veterinary literature on the subject he had failed to see it mentioned, therefore he had taken the liberty of naming and describing this disease. The disease is spoken of as "gong-lameness" in the fire department.

Drs. Walmer and Robinson made a report on some tuberculous herds recently tested with tuberculin. In a herd of forty-eight cows, three reacted. Two of these cows were in fair condition, large milkers. Reaction reached 108° and 107.6°. Each received 2 c.c. of tuberculin, and in both cases the milk-secretion was permanently stopped, and each cow had been milking three and one-half gallons a day. Postmortem alterations were almost microscopic in size, and carcasses passed for meat. A "bulling cow" in this herd, whose normal temperature was 102° F., went to 103° F. in fifteen hours. She was killed by request of the owner, but no diseased condition was found. Another cow in this herd had a large ulcerating tumor on the jaw, supposed to be actinomycotic in origin. This cow did not react. The tumor was examined by Dr. Lamb, of the Army Medical Museum, and proved to be tuberculous.

In another herd of thirty-one cows, fifteen reacted and were condemned for dairy purposes. The owner endeavored to have them slaughtered in the District of Columbia, but failed. He then shipped them to Alexandria, Va., but the efficient State Veterinarian, Dr. Niles, was notified, and he refused to let them be slaughtered for food purposes. They were then shipped to Baltimore, and Dr. Clement was notified, and he promptly turned them down. The owner then (March 8th) shipped these fifteen cows to Wilmington, Del., and they were there slaughtered and sold, since the State was without any official veterinarian who could be notified. This illustrates the value of official veterinarians acting in harmony and the loss a State bears without having one.

The recent Farmers' Institute held at Alexandria, Va., was brought up in this discussion, and the action of Major Alvord, Chief of the Dairy Division, Bureau of Animal Industry, was severely criticised, for denouncing the use of tuberculin in testing dairy-herds.

The question of passing cows for dairy purposes in which but three of the teats were secreting, was warmly discussed by those present. On most farms the owners claimed to use the milk of "three-teaters" for family purposes only. Dr. Robinson found pus in most of these non-secreting quarters. Where quarters were atrophied and clear of nodules and pus, he passed the cows for dairy purposes.

In this discussion the recent decisions of the attorney for the District was severely condemned, in which he held that cream was not milk, and that dairymen who have had their licenses revoked for keeping unsanitary dairies could ship cream into the District without having such license.

Meeting adjourned.

J. P. TURNER,
Secretary.

